# The influence of obesity, smoking, and serum follicular stimulating hormone in azoospermic patients on testicular sperm extraction-intra cytoplasmic sperm injection outcomes: A retrospective cohort study

**DOI:** 10.1097/MD.0000000000014048

**Published:** 2019-01-25

**Authors:** Guy Shrem, Yana Brudner, Yuval Atzmon, Mediea Michaeli, Adrian Ellenbogen, Einat Shalom-Paz

**Affiliations:** a Department of Obstetrics & Gynecology, Hillel Yaffe Medical Center, Hadera; b The Bruce and Ruth Rappaport Faculty of Medicine, Technion, Israel Institute of Technology, Israel.

**Keywords:** follicular-stimulating hormone, intra-cytoplasmic sperm injection, in-vitro fertilization, obesity, smoking, testicular sperm extraction

## Abstract

To examine the effect of serum follicle-stimulating hormone (sFSH) level, body-mass index (BMI) and smoking on Testicular Sperm Extraction–Intracytoplasmic Sperm Injection (TESE–ICSI), and pregnancy outcomes.

In this retrospective study, data were extracted from files of 52 azoospermic men who underwent TESE and in-vitro fertilization (IVF)-ICSI in our IVF unit. Demographic information, treatment cycle follow-up and pregnancy outcomes were collected.

Fifty-two patients underwent 79 TESE due to azoospermia in 143 IVF cycles. Smoking was found to significantly affect sperm motility in TESE specimens before freezing (45.5% vs 14.8%; *P* <.001); however, this finding did not influence the pregnancy rate. Male FSH was inversely correlated with testicle volume (r = −0.595, *P* <.0001). Body weight did not affect semen parameters after TESE or ICSI outcomes.

Among azoospermic patients with extremely poor sperm quality, male BMI, male FSH or smoking did not have an adverse effect sperm parameters or pregnancy and delivery rates.

## 1 Introduction

Absence of spermatozoa in the ejaculate (azoospermia) is found in 15% of infertile men. Surgical approaches combined with assisted reproductive technology (ART) provide fertility options for those couples. Azoospermia is classified as obstructive azoospermia (OA) or non-obstructive azoospermia (NOA). These conditions describe differences in the ability of the testis to produce and deliver spermatozoa.

Surgical methods have been developed to retrieve spermatozoa from the testicles in azoospermic men. Testicular sperm extraction (TESE)^[1]^ is the preferred surgical method for sperm retrieval from azoospermic men and is effective for both OA and NOA.^[2]^

Various lifestyle parameters can influence the sperm profile, including basal FSH levels, body-mass index (BMI), smoking, cause of male infertility, and age among the general population. However, the influence of these parameters on treatment outcomes of TESE–intracytoplasmic sperm injection (ICSI) is not clear.^[3,4]^

In this retrospective cohort study, we examined the effect of serum FSH levels, BMI, and smoking on TESE–ICSI and pregnancy outcomes.

## 2 Material and methods

### 2.1 Study participants

A cohort of 52 azoospermic men underwent TESE and in-vitro fertilization (IVF)–ICSI from 2009 through 2013 at the IVF unit at Hillel Yaffe Medical Center. The study was approved by the Intuitional Ethics Committee.

### 2.2 Patient evaluation

All patients diagnosed with azoospermia after 2 sperm analyses were evaluated for the etiology. The evaluation included physical examination by a urologist, hormonal profile, testicular ultrasound, karyotype, and Y-chromosome microdeletion.^[5]^

### 2.3 Testicular sperm extraction

TESE was conducted in our center under general anesthesia by a urologist, as described previously.^[6]^ Specimens were processed by an embryologist as follows. Six testicular biopsies were enzymatically digested with collagenase (0.8 mg/mL, Sigma) for 2 hours and centrifuged at 200 g for 10 minutes. The pellet was dissolved in 100 to 200 μL of Sperm Preparation Medium (Medicult, Aarhus, Denmark). Of this suspension, 20 μL was analyzed on a slide divided to 10 to 25 drops and evaluated under the microscope for the appearance of spermatozoa at magnification of 400. The total number of spermatozoa was calculated from the number per microscopic field or was counted per slide. Motility was documented as total motility and as a percentage. All testicular samples were cryopreserved. Ovarian stimulation was initiated only after sperm was retrieved.

### 2.4 Fertilization and embryo development

The ICSI procedure followed the TESE according to previously reported technique^[7]^ at 200 g at 10 minutes, and the pellet was suspended in 100 to 200 μL of sterile medium (Medicult). Two μL of this suspension was added to 3 μL drops of medium containing 0 to 3 μL of polyvinyl pyrrolidone in a sterile Petri dish covered in mineral oil. After the cells settled, each drop was examined for the presence of elongated spermatids by screening each microscopic field at 400X. A single spermatozoon was taken up into an ICSI pipette (Humagen, Charlottesville, SC) and transferred to a collecting drop. Motile and morphologically normal sperm were preferred, but when only immotile or abnormal sperm were found, these were used for standard ICSI. Maximum collection time did not exceed 4 hours. The selected sperm was found by embryologist at usual magnification of 200X to 400X. Conventional ICSI was performed using an inverted microscope. The injected oocyte was transferred to a 4-well dish containing either ISM1 medium (Origio) or Cleavage medium (Sage) pre-equilibrated culture media, overlaid with oil from the same company. The culture dish was incubated at 37°C to 37.2°C with 5.3% to 5.5% CO_2_.

Sixteen to 18 hours after ICSI, fertilization was assessed as previously described.^[8]^ Further embryonic development was evaluated daily. Embryo quality was assessed on day 2 or 3. Top-quality embryos on day 2 were defined as presenting with 4 equal size blastomeres and no more than 10% fragmentation. Top-quality embryos on day 3 were those with at least 7 equal blastomeres and no more than 10% fragmentation.

Pregnancy was determined by positive beta Human Chorionic Gonadotropin (βHCG) test 14 days after embryo transfer. Clinical pregnancy was defined as the presence of intrauterine gestational sac with fetal heart activity.

### 2.5 Data collection

Demographic data recorded included age, infertility diagnosis, and basal hormone levels for male and female partners. Treatment follow-up data included protocols, gonadotropins used, number of follicles and endometrial thickness during treatment, number of mature and immature oocytes retrieved, fertilization, and cleavage rate. The numbers of transferred embryos, implantation, and pregnancy rates, as well as pregnancy outcome, were also recorded. All viable intrauterine pregnancies were followed until delivery. Pregnancy and neonatal data were recorded, including gestational age at delivery, and any abnormalities.

### 2.6 Statistical analysis

Statistical analysis was performed using SPSS software package (SPSS Inc., Chicago, IL). We used Shapiro–Wilks test to evaluate the distribution of the data. Comparisons were analyzed using Student *t* test or Mann–Whitney *U* test, each when appropriate. Proportions were compared using Chi-square or Fisher exact test. *P* values less than .05 were considered significant. We used a multivariate logistic regression analysis model to rule out any other confounders that might influence the clinical results.

## 3 Results

Fifty-two patients underwent 79 TESE due to azoospermia in 143 IVF cycles. The demographic and clinical characteristics of the male patients are presented in Table 1 and for female partners in Table 2. Table 3 summarizes the ICSI cycles. There was a median of 7 oocytes per cycle, which yielded a median of 3 normal fertilizations (2PN), no top-quality embryos developed. The cumulative chemical pregnancy rate per TESE was 59.6%, the cumulative clinical pregnancy rate per TESE was 57.7%, with cumulative delivery rate of 44%. The pregnancy rate per IVF–ICSI treatment was 38.7% and clinical pregnancy rate per transfer was 32.4% with a 25.2% delivery rate.

**Table 1:**
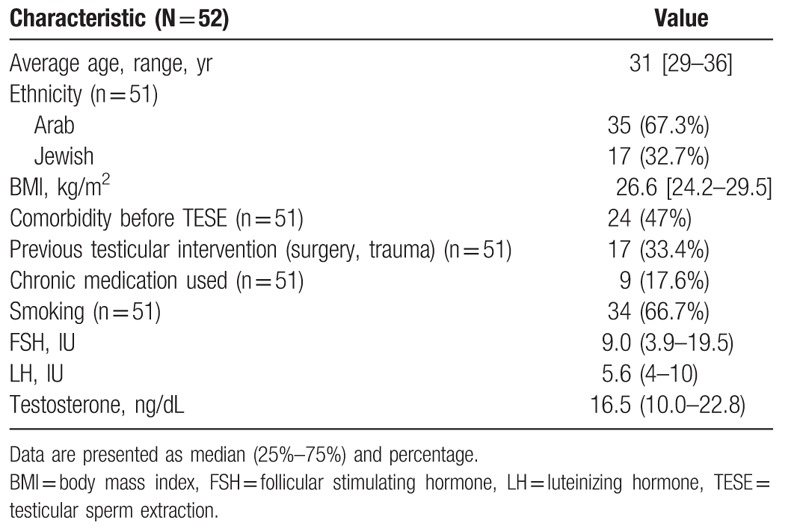
Characteristics of the male patients.

**Table 2:**
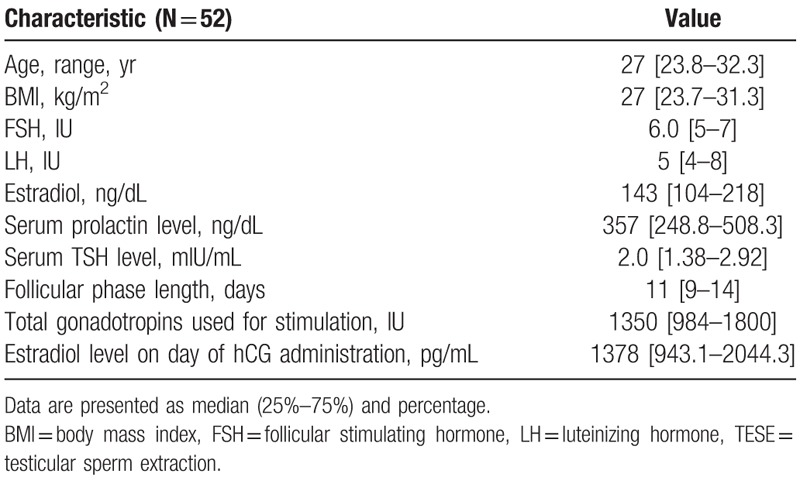
Characteristics of the female patients.

**Table 3:**
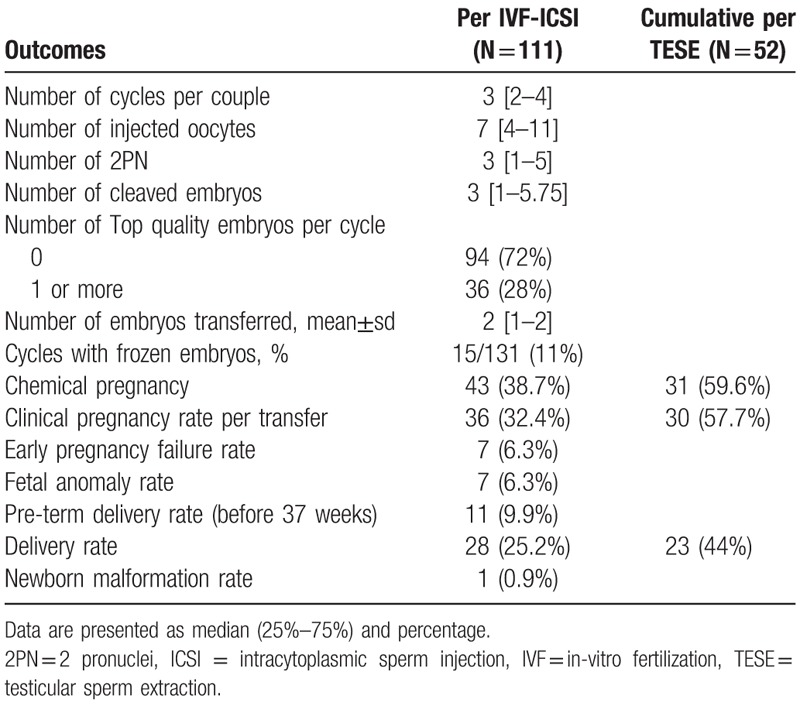
IVF-ICSI outcomes.

We divided the cohort into nonsmokers and smokers and found that the only significant difference was reflected in sperm motility after TESE before freezing (45.5% vs 14.8%; *P* <.001; respectively) (Table 4). However, smoking did not have a significant effect on pregnancy outcomes.

**Table 4:**
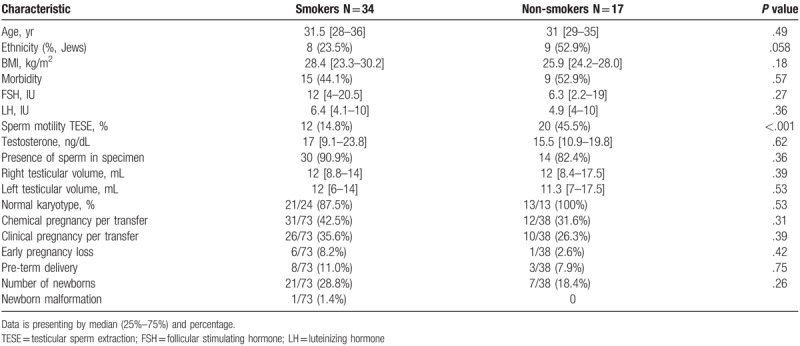
Characteristics and cycle outcome compared between smokers male and non-smokers.

Evaluating the influence of male-FSH on pregnancy outcomes per TESE cycles (total of 52 cycles), we found that lower FSH levels were correlated with positive pregnancy test and delivery rate (Table 5). A univariate analysis found that median male FSH 5 international units (IU) compared to median of 13.5 IU were significantly correlated with positive pregnancy rate and delivery rate (*P* = .018 for chemical pregnancy; *P* = .008 for clinical pregnancy) (Table 6).

**Table 5:**
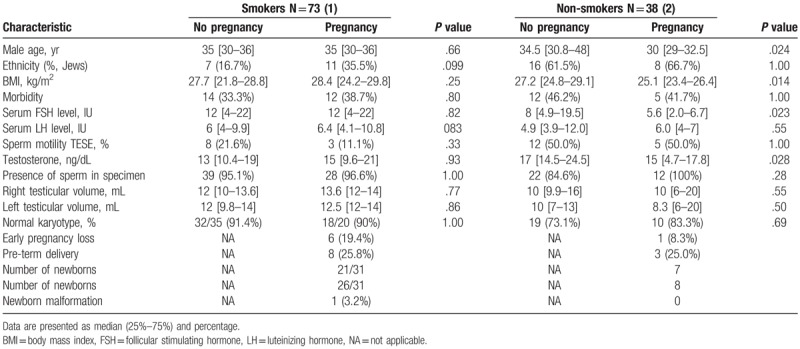
Pregnancy outcomes of smokers and non-smokers azoospermic patients.

**Table 6:**
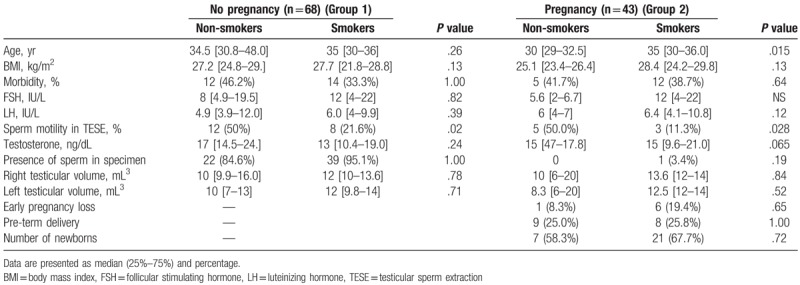
Comparison between pregnant to non-pregnant groups with sub-analyzing to smoking.

Univariate analysis of all IVF–ICSI cycles conducted for those couples we found that age less than 37 was significantly correlated with positive pregnancy outcome: chemical pregnancy 41% versus 15%, *P* = .039 and clinical pregnancy 35.6% versus 10%, *P* = .032.

Multivariable logistic regression model was used to assess the association between several independent parameters and pregnancy outcomes, including smoking, male FSH, male age, presence of motile sperm before freezing, and BMI. Motile sperm before freezing was significantly correlated with an increase in chemical pregnancy (OR = 4.26; *P* = .018; 95% CI 1.27–14.09). Advanced paternal age had a significant adverse correlation with live birth (OR = 0.86; *P* = .027; 95% CI 0.76–0.98).

Testicular volume in this model was not correlated with TESE or pregnancy outcomes.

There was 1 neonate with anomalies (5.5%).

## 4 Discussion

The present study evaluated the outcomes of azoospermic couples who underwent TESE–ICSI cycles. We focused on male age, smoking status, BMI, and FSH, predictive factors known to influence IVF outcome that is known to affect fertility outcomes among patients with very poor prognoses. To the best of our knowledge, this is the first study to evaluate the influence of these parameters in relation to TESE due to azoospermia.

It is well-established that fertility of both partners is adversely affected by smoking and smoking is associated with decreased IVF–ICSI success rates.^[9,10]^ Some studies identified smoking as reducing semen parameters.^[9–11]^ Lotti et al found that smokers had lower ejaculate and seminal vesicle volume as compared to non-smokers.^[12]^ Other studies reported that cigarette smoking has an overall negative effect on semen parameters, primarily sperm count and motility.^[13,14]^ Waylen et al reported significant evidence on the negative effect of cigarette smoking on ART clinical outcomes with 0.53 odds ratio for live birth per cycle and significantly higher odds (2.65) of spontaneous abortions.^[15]^

No previous study evaluated the impact of smoking on TESE results. The present study found significantly fewer motile sperm in the smokers compared to the non-smokers. However, this did not affect the pregnancy and delivery rates in our study. In non-TESE cycles, smoking was suggested to cause genetic damage due to tobacco and reactive oxygen species.^[16]^ The assumption is that toxins originating from cigarettes damage sperm mitochondrial activity and damage the chromatin structure in human sperm, therefore, leading to impaired fertilization capacity was not evaluated among azoospermic patients with very poor semen quality.^[17,18]^

BMI was also evaluated for its influence on male fertility. Evidence varies regarding male obesity and subfertility. Increased DNA damage to sperm, decreased mitochondrial activity, increased oxidative stress in testes, and poorer ART outcomes were suggested to be s influenced by obesity. Conflicting evidence is presented in studies discussing the influence of BMI on sperm quality but it was related to decreased ejaculate volume, lower sperm concentration, and increased DNA damage.^[4,19,20]^ Other studies reported no significant correlation between BMI and semen parameters.^[21]^ A meta-analysis by Le et al demonstrated that overweight or obesity in males had no negative influence on clinical pregnancy and live birth rates in ART.^[22]^ Our study did not demonstrate any effect of overweight on semen parameters after TESE or on ICSI outcomes.

Paternal age might have an effect on pregnancy outcomes in ART. Conflicting data have been presented regarding an association between increased paternal age and lower fertilization rates, decreased blastocyst formation, and pregnancy rates.^[23–26]^ Univariate regression revealed that paternal age above 37 years was significantly adversely correlated with chemical pregnancy (*P* = .032) and clinical pregnancy (*P* = .039). The sensitivity was 92.5%, but the specificity was 24.3% and this was not seen in multivariate regression.

Paternal FSH and testosterone in our study did not correlate with TESE outcomes. Other studies found that an FSH cutoff of 20 IU predicted pregnancy.^[27]^ Cissen et al developed a predictive model for obtaining spermatozoa with TESE In a retrospective, multicenter study, with data from 1371 TESE procedures they found that older male age, higher levels of serum testosterone and lower FSH and luteinizing hormone (LH) levels were predictive of successful sperm retrieval. They did not evaluate fertilization and pregnancy rates.^[28]^

This study had a few limitations, including the relatively small sample size and retrospective design. We included all azoospermic patients who underwent TESE, without reference to the etiology of azoospermia. Moreover, potential confounders include patient selection, and clinical and laboratory techniques used to find spermatozoa.

The strengths of this study include evaluation of the female partner and fertilization rates. The long term follow-up included pregnancy and delivery rates and neonatal outcomes.

In conclusion, this study evaluated the influence of different parameters on TESE–ICSI cycles. In those studied patients, as sperm quality is extremely low, no adverse influence on sperm parameters was seen. However, we advise our patients to maintain a healthy lifestyle, including normal weight and avoiding smoking, to prevent future health problems.

## Author contributions

All authors contributed substantially to this work. The authors collectively developed the original concept of this study. GS wrote the manuscript. ESP revised it critically. Data collection was performed by YB, GS, and YA, statistical analysis by ESP. All authors contributed to critical discussion and reviewed and approved the final version of the manuscript for submission.

**Conceptualization:** Guy Shrem, Adrian Ellenbogen, Einat Shalom-Paz.

**Data curation:** Guy Shrem, Yana Brudner, Einat Shalom-Paz.

**Formal analysis:** Guy Shrem, Einat Shalom-Paz.

**Investigation:** Guy Shrem, Mediea Michaeli, Einat Shalom-Paz.

**Methodology:** Guy Shrem, Einat Shalom-Paz.

**Project administration:** Guy Shrem, Einat Shalom-Paz.

**Resources:** Guy Shrem.

**Supervision:** Einat Shalom-Paz.

**Visualization:** Yuval Azmon, Einat Shalom-Paz.

**Writing – original draft:** Guy Shrem.

**Writing – review & editing:** Guy Shrem, Einat Shalom-Paz.

Guy Shrem orcid: 0000-0002-3825-3965.
